# The global limits and population at risk of soil-transmitted helminth infections in 2010

**DOI:** 10.1186/1756-3305-5-81

**Published:** 2012-04-26

**Authors:** Rachel L Pullan, Simon J Brooker

**Affiliations:** 1 Faculty of Infectious and Tropical Diseases, London School of Hygiene and Tropical Medicine, London, UK; 2 Kenya Medical Research Institute-Wellcome Trust Research Programme, Nairobi, Kenya

**Keywords:** Soil-transmitted helminths, *Ascaris lumbricoides*, *Trichuris trichiura*, Hookworm, Transmission limits, Urbanisation, Population at risk

## Abstract

**Background:**

Understanding the global limits of transmission of soil-transmitted helminth (STH) species is essential for quantifying the population at-risk and the burden of disease. This paper aims to define these limits on the basis of environmental and socioeconomic factors, and additionally seeks to investigate the effects of urbanisation and economic development on STH transmission, and estimate numbers at-risk of infection with *Ascaris lumbricoides*, *Trichuris trichiura* and hookworm in 2010.

**Methods:**

A total of 4,840 geo-referenced estimates of infection prevalence were abstracted from the Global Atlas of Helminth Infection and related to a range of environmental factors to delineate the biological limits of transmission. The relationship between STH transmission and urbanisation and economic development was investigated using high resolution population surfaces and country-level socioeconomic indicators, respectively. Based on the identified limits, the global population at risk of STH transmission in 2010 was estimated.

**Results:**

High and low land surface temperature and extremely arid environments were found to limit STH transmission, with differential limits identified for each species. There was evidence that the prevalence of *A. lumbricoides* and of *T. trichiura* infection was statistically greater in peri-urban areas compared to urban and rural areas, whilst the prevalence of hookworm was highest in rural areas. At national levels, no clear socioeconomic correlates of transmission were identified, with the exception that little or no infection was observed for countries with a per capita gross domestic product greater than US$ 20,000. Globally in 2010, an estimated 5.3 billion people, including 1.0 billion school-aged children, lived in areas stable for transmission of at least one STH species, with 69% of these individuals living in Asia. A further 143 million (31.1 million school-aged children) lived in areas of unstable transmission for at least one STH species.

**Conclusions:**

These limits provide the most contemporary, plausible representation of the extent of STH risk globally, and provide an essential basis for estimating the global disease burden due to STH infection.

## Background

Historically, soil-transmitted helminth (STH) infections were prevalent in parts of Europe, Japan, South Korea, Taiwan, the Caribbean and the southern states of America [1-6] but sustained control efforts and economic development helped to eliminate STH transmission from these countries [7-10]. In many parts of sub-Saharan Africa and South and Southeast Asia, there had, until recently, been little change in the prevalence of STH over the last half of the 20^th^ century [3,11,12]. In the last ten years, however, there has been increased political and financial support for the global control of STH infection, with a strong focus on school-based deworming [13]. Where scaling-up of treatment has happened it has occurred in changing social and economic contexts, including increased urbanisation [14,15]. Such a changing landscape necessitates accurate description of the contemporary distribution of STH transmission and population at risk, information which can inform the estimation of the global burden of disease due to STH [16].

Here, we present a detailed description of the global limits for *Ascaris lumbricoidesTrichuris trichiura* and the hookworms (*Ancylostoma duodenale* and *Necator americanus*) using surveys of infection prevalence derived from the Global Atlas of Helminth Infection (GAHI) [17], which are linked to high-resolution data on climatic and socio-demographic indicators, human population density and settlement patterns. The main aims of this paper are to (i) define biological limits of transmission suitability; (ii) investigate the influence of urbanisation and settlement patterns on levels of STH infection; and (iii) explore the modification of STH risk by economic development. Attention is focused on regional and species-specific differences in exclusions, with an emphasis on how best to define the limits of stable and unstable transmission. The resulting limits are subsequently used to estimate the total and school-aged populations at STH transmission risk in 2010.

## Methods

### Parasitological survey data

This analysis utilizes data on the prevalence of STH species collated in the GAHI (http://www.thiswormyworld.org) [18]. This project aims to provide an open-access, global information resource on the distribution of STH and schistosomiasis, with the specific aims of 1) describing the global distribution and prevalence of infection of each species and 2) highlighting geographical areas for which further survey information is required. The developed maps, along with sources of identified surveys, are presented on an open access website (http://www.thiswormyworld.org). The GAHI database is regularly updated using structured searches of the formal and grey literature, and has strict inclusion criteria: only random or whole community samples are included, excluding data from hospital or clinic surveys or surveys among non-representative sub-populations, such as among refugees, prisoners or nomads. For data from clinical trials or cohort studies, only baseline, pre-intervention estimates of prevalence are included. Efforts are made to geo-position each survey to a single longitude and latitude using a combination of electronic gazetteers, national school and village databases digitised from topographical maps and contact with authors who used a global positioning system (GPS; see Brooker et al. [18]). Where this point geo-position was not possible, efforts were made to geo-position surveys to the second administrative level, using the 2009 version of the Administrative Level Boundaries project (SALB) [19].

### Defining the biological limits of transmission

The purpose of this first analysis was to determine biological and climatic suitability for STH transmission, based on post-1980 data that could be geo-located to a single survey location. To reduce the influence of control measures, we excluded data collected after the initiation of national STH control programmes, defined as >25% of the at-risk school-aged population receiving anthelmintic treatment for five years prior to the survey date, as reported to the World Health Organization (WHO) [20]. This resulted in the exclusion of 746 survey points from Burundi, Egypt, Honduras, Lao PDR, Mali, Nepal, Niger, Peru, Sierra Leone, Uganda and Venezuela. Data were also excluded if surveys were conducted in an irrigated area, which was classified as districts with >50% land surface equipped for irrigation [21], excluding a further 25 survey points in Afghanistan, Egypt, India, Madagascar, Mexico, Nepal, Pakistan, South Africa, Thailand, Venezuela and Viet Nam. On the basis of the above, 4,633 independent prevalence surveys were included.

Experimental and field studies indicate that the survival and development of STH free living stages, and hence STH transmission, are crucially dependent on ambient and surface temperature and humidity [22-25]. Indirect estimates of these factors can be identified from high-resolution satellite and meteorological data. Synoptic mean, minimum and maximum monthly values of land surface temperature (LST) for the period 1950–2000 were derived from the WorldClim database of interpolated global weather station temperature data at 1 km spatial resolution [26,27]. Humidity was indirectly estimated on the basis of (i) annual precipitation rates available in the WorldClim database [26]; (ii) Potential Evapo-Transpiration (PET, a measure of the ability of the atmosphere to remove the water through evapo-transpiration); and (iii) Aridity Index (AI, calculated as mean annual precipitation divided by mean annual PET) [28,29]. In addition, extremely arid areas, such as deserts and their fringes, were identified using the GlobCover Land Cover product [30,31] for which the “bare areas” class denotes deserts.

The environmental and climatic data were imported into ArcMap 9.2 (ESRI, Redlands, CA) and linked by geographical location to the parasitological survey data. Analysis was stratified by region (Asia, Latin America and Africa plus the Middle East) and by climatic zone (tropics, sub-tropics/temperate) [32]. Upper and lower thermal and humidity constraints for each species were investigated using scatter plots and box plots. Two sets of biological limits were identified: areas that were biologically unsuitable (where median and mean observed infection prevalence was <0.1%); and areas with low/unstable transmission (where median and mean infection prevalence was <2%). The appropriateness of selected limits were subsequently assessed using box plots of point and district-level district estimates according to the limits and compared using a Kruskal-Wallis non-parametric test. Based on observed relationships, region and species-specific contour maps were developed on a 1 × 1 km grid to mask areas as biologically unsuitable for STH transmission, and areas where transmission is likely to be low/unstable. Districts were masked if >50% of their surface area was covered by at least one of the climatic masks. The identified limits were, however, not applied to irrigated areas.

### Investigating the effect of urbanisation and settlement patterns on STH risk

The extent by which population density and urbanisation impacts upon STH transmission is uncertain [33]. The purpose of this analysis therefore was to determine whether STH transmission risk varies according to population density or settlement patterns and whether risk should be modified according to urbanisation, as has been done previously for malaria [34]. Population density was estimated using a global population database (Global Rural Urban Mapping Project (GRUMP)) [35,36]. To ensure prevalences were contemporaneous with the population data, analysis was restricted to surveys conducted between 2005 and 2011. Survey locations were classified as urban using an updated 2010 urban extents (UE) mask derived from GRUMP. The remaining surveys were classified as peri-urban (<15 km from the UE edge and population density >250/km^2^), rural (>15 km from the UE edge and/or population density <250/km^2^), and extreme rural (population density <1/km^2^) using the Gridded Population of the World version 3 (GPW3) population database, projected to 2010 by applying national, median variant, urban and rural-specific growth rates per country [35,36].

The effect of urbanisation on infection prevalence was assessed by identifying spatially and temporally matched urban–rural pairs. Here each urban survey prevalence value was matched to surveys in peri-urban and rural areas that were conducted within a 100 km and five year window. When more than one survey originated from the same UE, the mean prevalence was calculated. Averages of the peri-urban and rural sets of surveys were calculated and assigned to their urban counterpart, generating a series of map-defined urban/peri-urban/rural matched pairs. As prevalence distributions were highly skewed, the prevalence values for matched pairs were compared using the Wilcoxon signed-rank test, and overall prevalence distributions by settlement type compared using the Kruskal-Wallis non-parametric test. For subgroup analysis, data were stratified by country-level developmental indicators: the proportion of the urban population with access to improved sanitation (<25%, 25–50%, >50%), and the GINI coefficient (<35%, 35–50%, >50%).

### Socioeconomic modification of risk at the country level

The third aim of this work is to determine whether territories can be identified as having no or very low STH transmission, or whether transmission risk should be modified, based on socioeconomic and development factors. Comparable, representative socioeconomic data are usually only available aggregated by country, and as such analysis of these limiting factors was restricted to the country level. National socioeconomic indicators were obtained from the World Bank databank [37], including GDP per capita; percentage of rural and urban populations with access to improved sanitation facilities; literacy rate in females ages 15–24 (proxy for maternal education), and GINI index (an indicator of the distribution of income within society, with 0% representing perfect equality and 100% perfect inequality) for the most recent year available. Parasite prevalence data were only included for the period 2005–2011, and incorporated all surveys conducted in this period that could be assigned to a country level. Scatter and box plots were used to explore ecological relationships between socioeconomic indicators and mean prevalence for each species.

### Estimating the population at risk of STH transmission

The identified biological and social limits were overlaid with the 2010 GRUMP population surface to estimate total population at risk (PAR) figures for each species individually, and at risk of infection with one or more species. To standardise to a single, representative age group of relevance to control, the proportion of the population of school-going age (5–14 years) was estimated for each country, based on the World Population Prospects: 2010 Revision Population Database [38].

## Results

### Defining the biological limits of STH transmission

**Figure 1:**
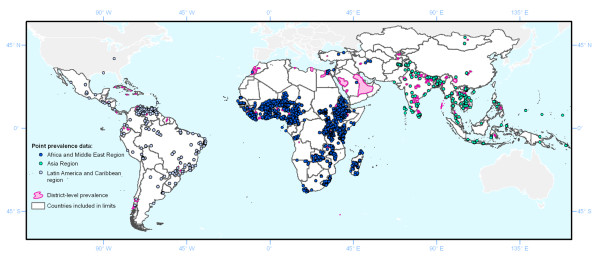
**Geographical distribution of available survey data geo-located to the point or district level**. Data were available for 4,840 unique survey locations, plus an additional 1,583 second administrative level areas.

Prevalence data for at least one helminth species were available for 138 countries: point-level survey data were available for 92 countries (representing 4,840 unique survey locations, 2,063 collected post-2004) and additional prevalence data were available at the second administrative level for a further 46 countries. Only 181 survey points were available for sub-tropical and temperate regions, representing 3.6% of the data. Thus, results presented here are restricted to stratification by world region only. Table 1 presents a more detailed breakdown of this data by region, and Figure 1 shows the geographical distribution of point and district data. The majority of the data comes from sub-Saharan Africa (SSA), but though fewer data are available for Asia and Latin America, the data that are available provide an adequate geographical spread of data points. Since few surveys were available for the North Africa and Middle East region, this area was combined with SSA.

**Table 1:** Summary of available STH survey data, by region

**Region**	**Total number of surveys available**^**a**^	**Unique survey locations**^**b**^	**Countries with point-level data**	**Surveys located to district level**^**c**^
Asia and Oceania	1,052	312	32	1174
Sub-Saharan Africa	4,536	4,215	37	207
Latin America and Caribbean	593	294	17	116
North Africa and Middle East	147	19	6	115

^a^ Includes surveys that can be located to a point, second administrative or country level.

^b^ Surveys geo-located to longitude and latitude.

^c^ Surveys geo-located to the second administrative level.

**Figure 2:**
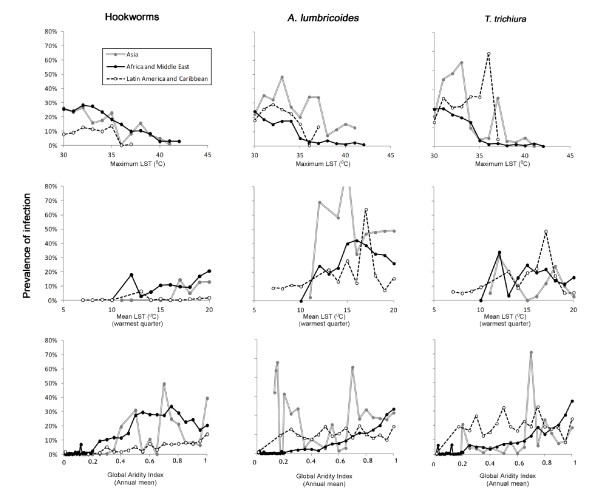
**Relationship between climatic factors and prevalence of STH infection.** Climatic factors estimated from interpolated weather station data, and prevalence of STH infection from 4,840 unique survey locations world-wide, stratified by world region. Estimates were derived for each survey point location and data for locations experiencing the same temperature are averaged for presentation.

Figure 2 shows the associations between climatic factors and prevalence, stratified by global region. Consistent with previous findings [33,39], clear relationships exist between prevalence of infection and LST for each species. For Africa and the Middle East, the prevalence of *A. lumbricoides* and *T. trichiura* is generally <4% in areas where maximum LST exceeds 35°C, and drops to <1% by 40°C. Hookworm infection remains prevalent throughout the upper end of the thermal range, only dropping to <2% when maximum LST exceeds 40°C. In contrast, relationships in Asia are less clear: although hookworm has similar limits to those seen in Africa and the Middle East, there is no clear upper thermal limit for *A. lumbricoides* in Asia, and prevalence of *T. trichiura* only drops below 1% at very high temperatures (>42^0^ C). There is no survey data for areas with maximum LST > 38°C for Latin America and the Caribbean. In contrast, hookworm appears more sensitive to low temperatures in all world regions, with observed prevalence <0.1% in those areas with mean LST below 10°C in the warmest quarter. Conversely, *A. lumbricoides* and *T. trichiura* infections remain prevalent throughout this lower thermal range.

All three species demonstrate similar relationships with aridity in Africa and the Middle East (Figure 2). Although there was considerable variation across semi-arid areas (aridity index >0.2–0.5), prevalence typically drops to less than 2% in areas classified as ‘arid’ (aridity index <0.2) and <0.1% in ‘hyper-arid’ areas (aridity index <0.03). This relationship is also seen for hookworm in other world regions. *A. lumbricoides* and *T. trichiura* are observed however, throughout much of the lower range of the aridity index in Latin America and the Caribbean, only dropping to <0.1% when the aridity index drops below 0.01. Interestingly, there appears to be no limiting relationship between *A. lumbricoides* and aridity in Asia. Comparison of the range of infection prevalence by land cover type, as classified by the GlobCover Project using high resolution satellite imagery, shows very large variation in the distribution of prevalence for all major land types (data not shown). Notably, whilst median infection prevalence is 0% for any STH in desert areas, for all three species at least 10% of the surveys conducted in areas classified as desert report infection prevalence of greater than 10%.

**Figure 3:**
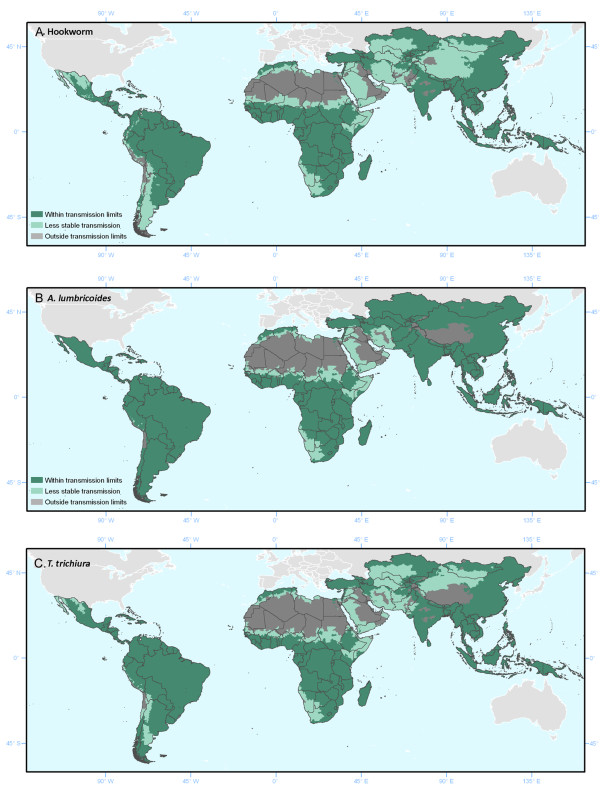
**Climatic suitability for (A) hookworm, (B)*A. lumbricoides*and (C)*T. trichiura*transmission defined by land surface temperature and aridity.** Areas were defined as stable (dark green), unstable (light green, where infection prevalence <2%), or no risk (light grey). Exclusion masks were developed in a step-wise fashion on the basis of species and region specific climatic thresholds.

**Figure 4:**
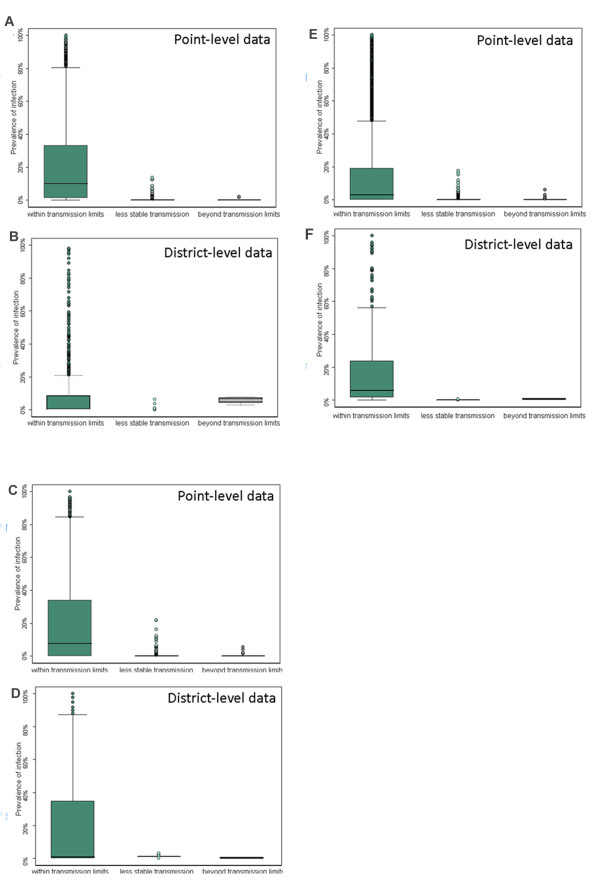
**Box and whisker plots of the distribution of point and district level data by transmission category**. Figures show hookworm **(A, B)**, *A. lumbricoides***(C, D)** and *T. trichiura***(E, F)**. The thick black line indicates the median, the box, the 25^th^ and 75^th^ percentile, whiskers indicate the range of the data and dots extreme outliers.

Based on the observed relationships, region and species-specific limits were developed and used to classify areas on the basis of climatic suitability as lying outside the plausible limits of transmission, namely unstable (median prevalence <2%) or no risk (median prevalence <0.01%). Districts (second administrative units) were then classified based on the most common transmission category by surface area. Figure 3 shows the spatial extent of transmission as defined by the transmission limits, described in Table 2. The distribution of point and district-level survey data within these limits is provided in Table 3 and Figure 4. For all three infections, there is strong evidence for difference in the distribution of survey data by transmission category: although wide variations in both point and district-level infection prevalence are observed within areas suitable for transmission, median infection prevalence falls below 0.001 in low transmission areas and is zero in areas classified as unsuitable for transmission. As can be seen from Table 3 and Figure 4, there is also strong evidence to support the discriminatory performance of the climatic masks when identifying districts were transmission is implausible.

**Table 2:** Climatic thresholds for region and species-specific transmission limits

	Africa and the Middle East		Asia		Latin America and the Caribbean	
	Unstable transmission	Beyond transmission	Unstable transmission	Beyond transmission	Unstable transmission	Beyond transmission
Hookworm	Aridity index^a^ <0.2	Max LST >42°C	Mean LST 5–10°C	Max LST >42°C	Max LST >36–40°C	Max LST >40°C
		Mean LST <10°C	Aridity index <0.2	Mean LST <5°C	Mean LST 10–15°C	Mean LST <10°C
		Aridity index <0.03		Aridity index <0.03	Aridity index <0.2	Aridity index <0.03
*A. lumbricoides*	Max LST >38–40°C	Max LST >40°C		Mean LST <10°C	Mean LST <5°C	Aridity index <0.03
	Aridity index <0.2	Mean LST <10°C				
		Aridity index <0.03				
*T. trichiura*	Max LST >38–40°C	Max LST >40°C		Max LST >42°C	Mean LST <5°C	Maximum LST >40°C
	Aridity index <0.2	Mean LST <10°C		Mean LST <10°C		Aridity index <0.03
		Aridity index <0.03				

^a^ Constructed using precipitation and evapo-transpiration; 0.03–0.2 = arid, <0.03 = hyper-arid; Max LST; maximum land surface temperature in the hottest month. Mean LST; mean land surface temperature in the warmest quarter.

**Table 3:** Distribution of helminth survey data, over the three transmission categories

	Hookworm			*A. lumbricoides*			*T. trichiura*		
	n	Median prevalence (range)	*P*^*a*^	n	Median prevalence (range)	*P*^*a*^	n	Median prevalence (range)	*P*^*a*^
Point-level survey data									
Within limits	5,157	0.1 (0, 1)		4,889	0.08 (0, 1)		4,891	0.03 (0, 1)	
Unstable transmission	228	0 (0, 0.14)		437	0 (0, 0.36)		436	0 (0, 0.16)	
Beyond limits	14	0 (0, 0.02)	<0.001	73	0 (0, 0.04)	<0.001	72	0 (0, 0.03)	<0.001
District-level survey data									
Within limits	1,351	0.001 (0, 1)		1,441	0.03 (0, 1)		1,419	0.06 (0, 1)	
Unstable transmission	32	0.001 (0, 0.06)		33	0.001 (0, 0.03)		29	0.001 (0, 0.02)	
Beyond limits	0	–	<0.001	25	0.001 (0, 0.04)	<0.001	21	0 (0, 0.01)	<0.001

^a^ Kruskal-Wallis non-parametric test.

On the African continent, areas of climatic non-suitability for all three species correspond to the extreme arid Sahara, masking out much of north Africa, whilst unstable transmission is expected for much of the arid Sahelian belt. The application of separate temperature masks for *A. lumbricoides* and *T. trichiura* widens the extent of the no risk and unstable transmission bands when compared with hookworm, masking the majority of Mali, Senegal, Burkina Faso, Chad and Republic of Sudan. Risk areas for all three species are however seen along the coast of Morocco, Algeria, Tunisia and Libya, and transmission cannot be excluded from irrigated districts in Egypt close to the Nile (representing 52% of Egypt’s districts). Unstable transmission associated with aridity is also seen in the Namib desert to the south, masking much of Namibia as well as western South Africa; in the horn of Africa (Somalia, Djibouti, Eritrea, northwest Kenya and eastern Ethiopia); and across the Red Sea in Yemen. Transmission is also precluded from the majority of the Arabian Peninsula, due to high temperatures and aridity. Transmission of *A. lumbricoides* can be ruled out for very few areas in Asia, although transmission is expected to be reduced in arid areas of the Gobi desert and Afghanistan, and in colder parts of the Himalayas. In contrast, hookworm and *T. trichiura* are masked from some pockets of central India (hookworm: 41 districts, *T. trichiura* 58 districts) and central Pakistan (hookworm: 34 districts, *T. Trichiura*: 25 districts), with reduced transmission expected throughout most of Pakistan (hookworm: 71 districts, *T. Trichiura*: 71 districts), central districts in India (hookworm only: 131 districts), mountainous areas of central Asia (including much of Uzbekistan and Kazakhstan) and in much of the northern part of the People’s Republic of China and Mongolia. Similarly, much of Latin American and the Caribbean can be considered climatically suitable for STH transmission, with transmission mostly limited by low temperatures or aridity. All three species are precluded from northern Chile, and hookworm from southern Chile and coastal regions of Peru, whilst reduced transmission of hookworm and *T. trichiura* is expected in more arid regions of Argentina.

### Investigating the effect of urbanisation and settlement patterns on STH risk

**Figure 5:**
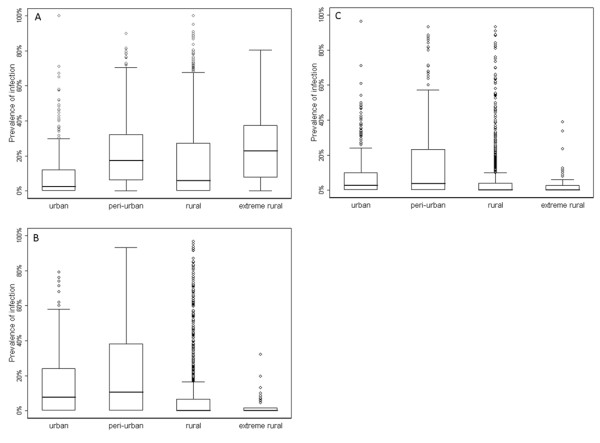
**Boxplots showing the distribution of helminth survey data by population density classification, for (A) hookworm, (B)*A. lumbricoides*and (C)*T. trichiura.*** Urban areas are defined using Urban Extents (UE) from the Global Urban Rural Mapping Project (GRUMP); peri-urban defined as <15 km from the UE edge and having population densities >250/km^2^, rural as >15 km from the UE edge and/or population density <250/km2 and extreme rural population density <1/km^2^ using an updated Gridded Population of the World (GPW3) population database. The thick black line indicates the median, the box, the 25^th^ and 75^th^ percentile, whiskers indicate the range of the data and dots extreme outliers.

Figure 5 presents box plots of the prevalence of STH species by population settlement type for 2,063 geo-located STH prevalence surveys conducted in 2005–2011. Although wide variation is evident for all three species across population density class, the results of the Kruskal-Wallis non-parametric test do suggest significant differences between groups at a global level (for all three infections *p* <0.001). For hookworm, urban infection prevalence is substantially lower than all other settings (median infection prevalence 2.4% vs. 7.7% in peri-urban and rural areas), whilst for *A. lumbricoides* and *T. trichiura* this situation is reversed, with higher infection prevalence observed in urban and peri-urban than in rural settings (8.2% vs. 0.0% and 3.0 vs 0.0% respectively). Findings were similar when results were stratified by world region, and by country urban development indicators (data not shown).

Matched urban–rural pairs were available for 57 urban centres in 19 countries. Results of Wilcoxen signed-ranks tests between infection prevalence values from the paired urban and rural sites are shown in Table 4. Results indicate that a strong difference exists between urban and rural hookworm prevalence (*p* < 0.001), although no difference is seen for *A. lumbricoides* and *T. trichiura*. This is emphasised when cities are stratified by both urban sanitation coverage and GINI: significant differences between urban and rural hookworm infection prevalence are seen when urban sanitation coverage exceeds 25%, and in more unequal societies (GINI >35%), suggesting that conditions in these cities are less conducive to hookworm transmission. For 12 cities, peri-urban paired data is also available (Brazil (1 city), Ethiopia (5), Gambia (1), Honduras (1), Kenya (1), Liberia (2) and Nepal (1)). Although there are insufficient data to allow stratification by development indicators, overall comparison of urban/peri-urban and peri-urban/rural matched pairs provides no evidence for a difference for hookworm, but does suggest that peri-urban infection prevalence is higher for both *A. lumbricoides* (Wilcoxen signed-ranks *p* = 0.03) and *T. trichiura* (*p* = 0.02).

**Table 4:** Results of Wilcoxon signed-ranks tests on prevalence values for each STH species, between GRUMP-UE defined urban (U) and rural (R) survey pairs

		**Number of pairs**			
		**Total**	***U ≥ R***	***U < R***	***p***^**j**^
**Global**^**a**^					
Hookworm		57	22	35	<0.001
* A. lumbricoides*		57	32	25	0.5
* T. trichiura*		57	26	31	0.5
By urban sanitation coverage ^b^					
Hookworm	<30% ^c^	10	5	5	0.8
	30-50% ^d^	39	14	25	<0.001
	>50% ^e^	8	3	5	0.06
* A. lumbricoides*	<30% ^c^	10	3	7	0.3
	30-50% ^d^	39	25	14	0.9
	>50% ^e^	8	3	4	0.6
* T. trichiura*	<30% ^c^	10	4	6	0.5
	30-50% ^d^	39	18	21	0.6
	>50% ^e^	8	3	4	0.7
By GINI coefficient ^f^					
Hookworm	<30% ^g^	14	6	8	0.1
	30-50% ^h^	37	12	22	0.005
	>50% ^i^	6	1	5	0.05
* A. lumbricoides*	<30%	14	8	6	0.5
	30-50% ^h^	37	22	15	0.5
	>50% ^i^	6	2	4	0.1
* T. trichiura*	<30% ^g^	14	5	9	0.9
	30-50% ^h^	37	18	19	0.4
	>50% ^i^	6	3	3	0.5

^a^ Matched pair data are available for 50 cities in Africa, two cities in Asia and three cities in Latin America.

^b^ Urban sanitation coverage refers to the percentage of urban population with at least adequate access to sanitation (protected pit latrines and upwards) in 2008. Source: World Bank Databank.

^c^ Includes cities in Burundi (1) and Ethiopia (12).

^d^ Include cities in Argentina (1), Burkina Faso (1), Gambia (2), Ghana (4), India (1), Kenya (1), Liberia (5), Mali (11) Nepal (1), Nigeria (2), Sierra Leone (1), Tanzania (1), Uganda (5) and Zambia (1).

^e^ Includes cities in Argentina (1), Brazil (1), Gambia (2), Honduras (3) and Zambia (1).

^f^ GINI coefficient is an indicator of the distribution of income within society with 0% representing perfect equality and 100% perfect inequality. Source: the World Bank databank (http://data.worldbank.org/).

^g^ Includes cities in Ghana (4) Liberia (1) and Sierra Leone (1).

^h^ Include cities in Burkina Faso (1), Burundi (2), Ethiopia (12), India (1), Kenya (1), Mali (11), Nepal (1), Nigeria (2), Rwanda (2), Tanzania (1) and Uganda (5).

^i^ Includes cities in Brazil (1), Honduras (3) and Rwanda (2).

^j^ Wilcoxon signed-ranks tests.

### Socioeconomic modification of risk at the country level

**Figure 6:**
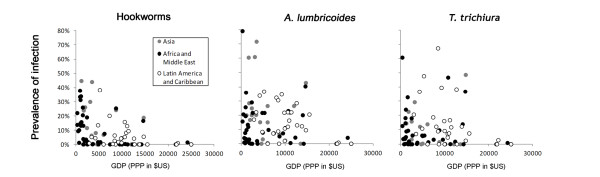
**Relationship between national gross domestic product and mean country infection prevalence, stratified by species.** Mean prevalence is calculated from surveys conducted 2005–2011. GDP (per capita in US$) for 2010; Gross Domestic Product per capita (current US$).

Analysis of the correlation between socioeconomic factors and infection prevalence at a country level was restricted to 109 countries (representing 2,407 unique surveys) with data collected between 2005–2011. Perhaps unsurprisingly, no clear association was seen between mean survey prevalence (2005–2011) and the proportion of households with access to improved sanitation or literacy rates in women aged 15–24 years (a proxy for maternal education) at a country level for any species (data not shown). Although very little contemporary survey data exists for those higher income countries that fall within areas climatically suitable for transmission (which includes much of the Caribbean, and several islands in Oceania), very low infection prevalence for all three countries is seen in those countries with GDP > US$ 20,000 in 2010, suggesting that this may provide a realistic cut-off (Figure 6). This excludes 33 of the 166 potentially endemic countries, representing only 1.4% of the potentially at-risk population.

### Total populations at risk

**Figure 7:**
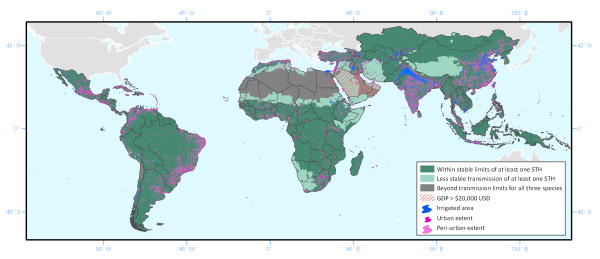
**Distribution of soil-transmitted helminth (STH) in 2010, applying climatic exclusion limits.** Indicating areas at stable risk of transmission for at least one STH species (dark green), unstable transmission of at least one species (light green) or at no risk of transmission for any STH species (dark grey). Pink hatching indicates countries excluded on the basis of socioeconomic status; also shown are irrigated areas (blue), urban extents (dark pink) and surrounding peri-urban extents (light pink).

Figure 7 presents the combined extends of STH transmission, highlighting those countries excluded on the basis of socioeconomic status. Also highlighted are irrigated areas (which may still support transmission even when climatic conditions are limiting), and the extents of urban and peri-urban areas in 2010, defined using GRUMP urban extents and population density surfaces.

The estimated total and school-aged 2010 PAR within areas of assumed unstable (parasites prevalence < 2%) and stable transmission globally and by world region are presented in Table 5. We estimate that 5.08 billion people (1.0 billion of school-going age) live in areas of stable hookworm transmission worldwide, 22% located in Africa and the Middle East, 69% in Asia and 9% in Latin America and the Caribbean (LAC). Similarly, 71% of the 5.3 billion people at risk of stable transmission with *A. lumbricoides* or *T. trichiura* live in Asia and Oceania, 18% in Africa and the Middle East, and only 11% in LAC. Within these stable limits, 25% live in urban areas (21%, 24% and 42% in Africa and the Middle East, Asia and LAC, respectively) and a further 24% in peri-urban areas, and so are likely to experience differing transmission risks from those in surrounding rural areas. Globally, an additional 143 million (31 million of school going age) live in areas of unstable transmission for at least one STH species. These areas, which represent 15% of areas classified as suitable for STH transmission, typically coincide with arid, low population density areas.

**Table 5:** Population at risk of STH infection in 2010

			**Population at risk (PAR) of infection (millions)**							
**Region**	**Total Pop(millions)**	**Excluded pop**^**a**^ **(millions)**	**Hookworm**		***A. lumbricoides***		***T. trichiura***		**Any STH species**	
			**Stable**^**b**^	**Unstable**^**c**^	**Stable**^**b**^	**Unstable**^**c**^	**Stable**^**b**^	**Unstable**^**c**^	**Stable**^**d**^	**Unstable**^**e**^
**Total population**										
***Global***	***5,634.9***	***78.3***	***5,076.6***	***269.4***	***5,231.3***	***184.5***	***5023.3***	***284.0***	***5,340.1***	***143.4***
SSA	864.7	0.08	810.5	48.9	715.7	98.8	715.7	98.8	810.5	48.9
NA/ME	447.4	38.6	268.3	83.7	254.3	85.2	254.3	85.2	268.3	83.7
Asia and Oceania	3,748.3	36.5	3,493.8	79.4	3,686.4	10.4	3,493.8	84.3	3,686.4	10.4
LAC and Caribbean	589.4	3.11	503.9	57.5	574.8	0.4	559.3	15.7	574.8	0.4
**School-aged (5–14 years) population**										
***Global***	***1078.9***	***12.5***	***966.1***	***55.5***	***990.7***	***43.2***	***948.5***	***62.7***	***1019.1***	***31.1***
SSA	228.6	0.01	214.4	12.8	189.0	26.4	189.0	26.4	214.4	12.8
NA/ME	87.1	7.35	50.5	16.4	47.6	16.8	47.6	16.8	50.5	16.4
Asia and Oceania	653.2	4.68	607.3	15.2	646.7	0	607.3	16.6	646.7	1.8
LAC and Caribbean	110.0	0.45	93.8	11.1	107.4	0.08	104.6	2.9	107.4	0.08

^a^ Population in countries with 2010 GDP (PPP) > US$ 20,000 are presumed to be at little or no risk of infection with any species of STH.

^b^ Stable transmission based upon region and species-specific climatic transmission limits.

^c^ Unstable transmission (median prevalence <2%) based upon region and species-specific climatic transmission limits.

^d^ Stable transmission of at ≥1 STH species.

^e^ Unstable transmission of at ≥1 STH species.

## Discussion

Here we provide a robust, contemporary and empirical description of the global limits of STH transmission, based upon comprehensive analysis of a suite of spatially-referenced environmental and socio-demographic data and STH prevalence data abstracted from the GAHI. Use of plausible climatic constraints upon transmission, based upon long-term temperature and rainfall data, provides improved spatial precision of limits and categories of risk, whilst consideration of socioeconomic factors increases plausibility at national levels. We estimate that there were 5.3 billion people at risk of stable transmission with one or more STH worldwide in 2010, 69% of whom live in Asia and 49% in urban or peri-urban environments. This concentration of population at risk in these areas reflects the relative distribution of population in the world.

The observed limiting relationship between all three STH species and climatic factors in Africa and the Middle East corroborate previous experimental and observational findings that transmission is implausible in extremely hot, cold or arid conditions [22-25,33,40], and likely reflects the dynamic processes involved in STH transmission, such as free-living infective stage development and survival [41]. Relationships are less clear in Asia, where there is substantially more seasonal climatic variation. This is especially true for *A. lumbricoides,* for which positive survey data exists even in extremely hot and arid regions of India and Pakistan. In contrast to other STHs, *A. lumbricoides* eggs can remain viable in soil for several months [41] and are more resistant to extreme temperatures [23], whilst *A. lumbricoides* larvae can undergo arrested development in the human body for several months [33], all features that may allow seasonal transmission of *A. lumbricoides* in environments that are hostile for much of the year. Limits for Latin America are less robust, as very few data were available for extreme environmental settings from this region.

This analysis also aimed to investigate modification of risk in urban areas. It is commonly assumed that hookworm is found more often in rural areas, whereas *A. lumbricoides* and *T. trichiura* are more prevalent in urban environments, an assumption only in part supported by the few studies that have specifically compared socioeconomically equivalent urban and rural populations [42-48]. In order to avoid subjectivity in the designation of urban/rural status, we have adopted an approach first proposed by Tatem *et al.* (2008), stratifying geo-located prevalence surveys by settlement type on the basis of population density using gridded population maps [34]. Our results provide strong evidence to suggest that urban areas experience significantly reduced hookworm prevalence, although substantial transmission can still occur. Interestingly, there is also some limited evidence that *A. lumbricoides* and *T. trichiura* prevalence is greater in peri-urban slum areas. Although the precise reason for the differing relationships by species is unclear, it is possible they may reflect the relative influences of socioeconomic status, sanitation, overcrowding and hygiene behaviours. However, it is important to emphasise the substantial heterogeneity in these findings. For instance, in 20% of matched pairs, urban hookworm prevalence was greater than surrounding rural prevalence, whilst globally the range in urban hookworm prevalence was seen to vary between nil and 90%. Given that an estimated 42% of the 1.15 billion population at risk in India alone live in peri-urban urban areas, it is clear that further work is needed to investigate the impact of increasing rapid urbanisation on STH prevalence.

Although conservative, these limits come with some major caveats. First, it should be emphasised that these results are based on an opportunistic sample of STH endemic countries, and as such do not all derive from nationally representative, spatially random surveys. However, whilst it is possible that their coverage might be subject to bias towards more wormy areas, this is less of a concern given that 2,722 (56%) of surveys were negative for at least one species, and 562 (12%) were negative for all three. Second, in defining these limits, we have implemented crude rules at global scales, and it is likely that at a sub-national level there will be some important anomalies. This is particularly the case for socioeconomic disparities in risk. Perhaps unsurprisingly, simple correlations between infection prevalence and indicators of development presented here failed to reveal any clear relationships, other than the absence of STH infection in those few countries with GDP per capita > US$ 20,000. Nevertheless, it has been argued that poverty reduction and improvements in hygiene and sanitation have played an important role in both the historical elimination of infection in the American South and other temperate areas [49], and the current rapid declines observed in middle-income countries including the People’s Republic of China and Brazil [50,51]. As such, it is likely that affluent communities living within climatically plausible limits will experience negligible exposure to infection. In the absence of global, fully comparable sub-national socioeconomic data, and given the risks of ecological bias when making such inferences, it is not possible to exclude such populations from global limits at this time.

A recent meta-analysis has revealed that adequate sanitation is associated with a reduced risk of STH transmission (between 40 and 50% reduced odds of infection) at a community level, although available data were limited in quality and scope [52]. The lack of association between STH risk and water and sanitation in the present analysis is probably due, in part, to a lack of detailed sub-national data on access to water and sanitation. There are however a number of new initiatives to map and monitor the coverage of adequate water and sanitation, both in communities [53] and in schools [54], which will provide a crucial information resource for future investigation of the changing STH distributions over time and the roles that water and sanitation play.

The next critical step in this planned series of work is to model variation in prevalence of infection, and of associated morbidity, within these defined limits. Previous global burden estimates for STH have attempted to capture geographical heterogeneity by assuming prevalence distributions within countries to be approximately normal [55,56]. This approach, which is severely constrained by scarce survey data, is unable to identify the location of high prevalence communities; neither is it able to account for heterogeneity in the distribution of human populations. These limitations however can be overcome by Bayesian model-based geostatistical (MBG) approaches, which can be used to predict the continuous distribution of disease outcomes, whilst fully accounting for the constraints of limited sampling, uneven data and spatial dependency [57,58]. MBG modelling approaches incorporating environmental covariates have been used successfully to estimate the burden of STH infections at country [59] and regional [60,61] levels, and have been applied at global levels for malaria [62]. As infection prevalence responds to increased coverage of large-scale deworming programmes, however, the use of traditional environmental covariates might prove less effective for predicting the distribution of STH. It is also likely that local endemicity may be substantially modified by changes in modern land use such as irrigation [63-65] and urbanisation [42]. As such, there is a pressing need to robustly quantify the influence of these human factors on the distribution of human helminth infections.

## Conclusion

In conclusion, results presented here clearly demonstrate that high and low LST and extreme arid environments limit transmission of STH species, and also suggest differing transmission dynamics in rural, peri-urban and urban environments. Whilst uncertainties remain about the precise global limits of STH transmission, most noticeably for areas of Asia, we have reduced them as far as possible using empirical associations with climatic and socioeconomic factors, and suggest that globally, 77% of the world’s population lived in areas of risk of stable transmission in 2010. Urgent attention is now needed to better define the distribution of STH risk within these limits, in particular addressing relationships between urbanisation, sanitation and infection. In addition to better quantifying the global burden of STH infection, this will allow the international community to better define priority populations for preventive chemotherapy, provision of improved water and sanitation and health education, thus providing cost-effective, targeted strategies for control.

## Competing interests

The authors declare that they have no competing interests.

## Authors’ contributions

RLP participated in the design of the study, carried out the analysis and drafted the manuscript. SJB participated in the design of the study, contributed to data assembly and helped draft the manuscript. Both authors read and approved the final version of the manuscript.
